# Application value of MRI-guided wire localization to the non-palpable breast lesions only shown in Breast MRI

**DOI:** 10.3389/fonc.2024.1325362

**Published:** 2024-05-24

**Authors:** Jiaqi Ma, Leina Hou, Xiufen Liang, Bin Yan, Qiang Dai, Yunmei Wang, Hongbian Gao, Jiang Zhu, Canxu Song, Quan Yuan

**Affiliations:** ^1^ Department of Radiology, Shaanxi Provincial Cancer Hospital, Xi’an, Shaanxi, China; ^2^ Department of Anesthesiology, Shaanxi Provincial Cancer Hospital, Xi’an, Shaanxi, China; ^3^ Department of Medical Oncology, Shaanxi Provincial Cancer Hospital, Xi’an, Shaanxi, China; ^4^ Department of Pathology, Shaanxi Provincial Cancer Hospital, Xi’an, Shaanxi, China; ^5^ Department of Breast Cancer, Shaanxi Provincial Cancer Hospital, Xi’an, Shaanxi, China; ^6^ Department of Ultrasonography, Shaanxi Provincial Cancer Hospital, Xi’an, Shaanxi, China

**Keywords:** MRI, breast cancer, non-palpable breast lesions (NPBL), MRI-guided, wire localization

## Abstract

**Introduction:**

Magnetic resonance imaging (MRI)-guided wire localization can be applied to assist to remove suspected breast lesions accurately. This study aimed to evaluate the clinical application value of this technique in Chinese women.

**Methods:**

A total of 126 patients (131 lesions) who had underwent such technique in our hospital from April 2017 to June 2023 were enrolled. 1.5T MRI system and a wire localization device were used. Image characteristics, clinical features and postoperative pathology were collected and analyzed.

**Results:**

All of 126 patients (131 lesions) were successfully localized by MRI and excised for biopsy. There were 39 malignant lesions (29.77%) and 92 benign lesions (70.23%). There was no significant correlation between the morphology of DCE-MRI and the ratio of malignant lesions (P=0.763), while there was a statistical correlation between the BPE, TIC curve and the malignancy rate (P<0.05). All the lesions were assessed according to BI-RADS category of MRI (C4A=77, C4B=40, C4C=12, C5=2). The malignancy rates were as follows: 16.88% for 4A lesions (13/77), 37.50% for 4B lesions (15/40), 75.00% for 4C lesions (9/12) and 100% for 5 lesions (2/2). There was a significant correlation between the BI-RADS category and the incidence of benign-to-malignant lesions (P<0.001).

**Conclusion:**

MRI-guided wire localization can assist to remove suspected breast lesions early, safely and accurately. This technique makes up for the deficiency of X-ray and ultrasound, improves the accuracy of diagnosis and resection therapy in intraductal carcinoma and early invasive carcinoma, and helps to improve the the prognosis of breast cancer.

## Introduction

Breast cancer is the most common malignant tumor in the world with a higher incidence than lung cancer. In 2020, there were approximately 2.26 million new breast cancer patients in the world, while there were 0.42 million new cases in China. Breast cancer has the highest increase rate in malignant tumors in Chinese women, which poses a serious threat to women’s life and health (1). Medical imaging is an important method for the early screening and diagnosis of breast cancer. According to the recommendations of the European Society of Breast Cancer Specialists (EUSOMA), magnetic resonance imaging (MRI) is used increasingly for breast cancer screening, detection, and staging (2). Breast MRI is useful for the detection of small lesions due to its high sensitivity, especially for curable early invasive cancer. However, the high sensitivity may limit its specificity to a certain degree (3, 4). It is undeniable that accurate tissue biopsy and pathological diagnosis are crucial for early screening.

Some suspicious lesions with negative palpation can only be detected by MRI, which are negative in x-ray and ultrasound (US) examination. Thus, MRI-guided biopsy is the only feasible method for clear diagnosis. MRI-guided breast biopsy has been fairly applied in Western countries, and some developed countries have required this technology to be essential for breast MRI examination (5, 6). Asian women have a smaller breast size and a higher percent breast density compared to Caucasian women (7–9). The American College of Radiology Breast Imaging Reporting and Data System (BI-RADS) divided breast structure into four categories: A, B, C, and D, which provided the risk for developing breast cancer to clinicians (10). The C and D types are considered to have denser breast structures. Women with breast density of 75% or greater percent of had four to six times higher risk of breast cancer compared with women with density in less than 10% (11, 12). Therefore, a dense structure is a strong risk factor for breast cancer. Based on the differences in breast structure, we speculate that the results of MRI-guided biopsy in Chinese women may be different from those in Caucasian women, as there are still limited reports about related research in China. In recent years, MRI-guided breast biopsy has been gradually developed in China, but there are still limited reports about related research (13, 14).

Based on the current situation, the results of MRI-guided wire localization and resection biopsy, which have been applied to 131 breast lesions in 126 female patients, were reported and analyzed.

## Materials and methods

### Patients

This retrospective study enrolled 131 lesions of 126 patients who underwent breast MRI-guided wire localization surgery at Shaanxi Cancer Hospital from April 2017 to June 2023. All lesions were detected by MRI, which showed no suspicious signs by primary first eye ultrasonography and Full Field Digital Mammography (FFDM). There were a total of 92 patients (94 lesions) with no suspicious findings based on the second eye ultrasonography, while the remaining 34 patients (37 lesions) refused such reexamination. The Ethics Committee of Shaanxi Cancer Hospital approved to waive the informed consent of patients in this study.

### Diagnostic MRI examination

The entire group consisted of 126 patients. A total of 29 patients were scanned by the 1.5-T MRI system (Toshiba, VISART, Japan), and 97 patients were scanned by the 3.0-T MRI system (Siemens, Magnetom Skyra, Germany). The patients were prone and the breasts were positioned within a dedicated surface breast coil with eight channels. All the patients signed the informed consent form before MRI scan. MRI scan sequences were as follows: axial T1WI/FSE and T2WI-STIR. Dynamic contrast-enhanced MRI (DCE-MRI) employs rapid, small-angle excitation of 3D dynamic sequences for fat inhibition in T1WI axial scanning, T1-weighted 3D FS gradient-echo images (five stages). A dose of 0.2 mL/kg of gadolinium diethylenetriamine (Gd-DTPA, Kang Chen, Guang Zhou, China) was injected through the cubital vein with a high-pressure syringe at a velocity of 2.0 mL/s. Fifteen milliliters of 0.9% saline was then injected with the same velocity to flush the vein. The scanning parameters of the 1.5-T DCE-MR are as follows: TR/TE 5.5/2.5 ms, FOV 300×210 mm, layer thickness 2.5 mm, layer spacing 0, and matrix 172×256. The scanning parameters of the 3.0-T DCE-MR are as follows: TR/TE 4.5/1.69 ms, FOV 360×360 mm, layer thickness 1.5 mm, layer spacing 0.3 mm, and matrix 448×524.

We used subtraction technology during image processing. All MRI images were assessed by two radiologists (with 10 and 30 years of experience in breast imaging diagnosis respectively). Radiologists analyzed the MRI signal, morphology, range, and enhancement characteristics of lesions, as well as the time–signal intensity curve (TIC). Radiologists who were informed about the patients’ clinical courses and imaging history such as FFDM, ultrasonography, and examinations of the breasts, evaluated MRI findings according to the BI-RADS for MRI (American College of Radiology, 2013) (15). All lesions were classified as BI-RADS Class 4 or above.

### MRI-guided wire localization

All patients were subjected to MRI-guided wire localization within 2 days to 2 months after diagnostic MRI scanning. The pre-MRI scanning and wire localization were performed within the same menstrual cycle. The equipment consists of the 1.5-T MRI system (Toshiba, VISART, Japan), a breast phased array coil with eight channels, and a special positioning device. The wire is specialized for MRI breast localization, which is produced by Bard Company in the United States (Model:479201, 20G× 90mm). The patient lay prone on the positioning frame, and the affected breast was fixed between a compression plate and a grid plate. Based on the principle of the closest distance between body surface and lesions, the operator punctured the breast from the lateral or medial position.

Compared with the previous MR image, DCE-MRI scanning was performed on the axial position (one to two stages) to identify the lesion location. Once the suspected lesion appeared, the scanning was immediately stopped and the image was sent to the post-processing workstation for calculation to obtain the needle mesh, aperture, and depth. After disinfecting the local skin, the operator inserted a guide wire locator into a suitable mesh (6×6 channels, diameter 2 mm, and adjacent spacing 1 mm), and then punctured breast in the correct hole. MRI axial rescanning was used to confirm that the end of the needle tip is located at the lesion. If the position is accurate, the hook was released, the sheath was removed, and the positioning device was evacuated. The last MRI axial and sagittal scan was performed to show the hook position. Finally, the surgeons chose an appropriate surgical path on the basis of FFDM, which displayed the spatial relationship between the wire and the lesion. The pathological diagnosis was completed by two pathologists with 10 and 20 years of work experience.

### Data collection and statistical analysis

The data include age, menstrual status, medical reasons, breast MRI signs and BI-RADS, modus operation, and pathologic diagnosis. SPSS version 21.0 (IBM, Chicago, USA) was used, and the chi-square test was the main method used. The two-tailed *p* < 0.05 was set as the limit of statistical significance.

## Results

MRI-guided wire localization was all successfully performed in 131 lesions of 126 patients (100%, 131/131). The patients’ age ranged from 26 to 70 years old, with a median age of 48 years. All lesions were successfully localized at once, and the time required for MRI-guided wire localization varied from 15 to 40 min. We recorded needle insertion distances ranging from 1.5 to 5 cm. Only six patients experienced slight bleeding at the acupuncture point, which stopped after brief compression. No patient has experienced needle displacement, breakage, hematoma, or infection at the puncture site. A total of 121 patients underwent single-lesion localization on unilateral breast, 3 patients underwent localization of bilateral breasts, and 2 patients underwent two-lesion localization in the single breast. All lesions were resected completely and all patients were diagnosed with clear pathological results. Subsequently, 19 patients underwent further surgical treatment due to the malignant biopsy results. The clinical information is shown in **Table 1**.

**Table 1:** Clinical characteristics of 126 patients.

Clinical characteristics	Number (%)
Age	
≤40	38 (30.16%)
40–60	81 (64.28%)
≥60	7 (5.56%)
Menstruation	
Premenopausal	92 (73.02%)
Postmenopausal	34 (26.98%)
Medical history	
Family history of breast cancer	15 (11.90%)
Postoperative unilateral breast cancer	9 (7.14%)
Ipsilateral breast cancer	15 (11.90%)
Contralateral breast cancer	5(3.97%)
History of chest radiation therapy	2 (1.59%)
History of gynecological cancer	5 (3.97%)
History of atypical breast lesions	25 (19.84%)
History of thyroid cancer	6 (4.76%)
Physical examination	44 (34.92%)

**Figure 1:**
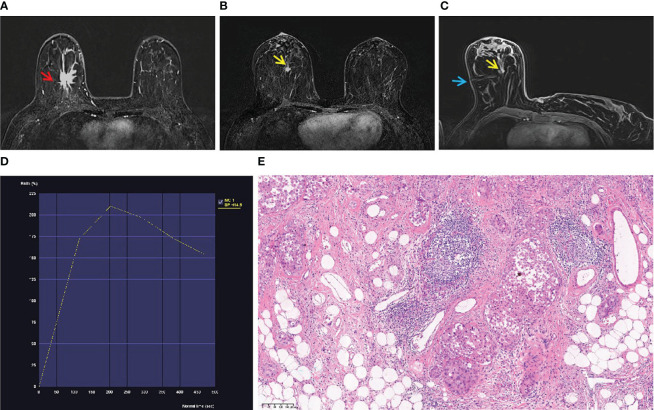
A 53-year-old woman with palpable mass in the right breast. **(A)** An irregular mass (red arrow) located in the right breast (BI-RADS 5) was shown in T1-weighted fat-suppressed contrast-enhanced MRI. It was confirmed as invasive breast cancer by puncture pathology. **(B)** There was a focal lesion (yellow arrow) below the mass in the right breast, and the BI-RADS category was considered as 4B. It was not found by ultrasound or mammography. **(C)** The lesion (yellow arrow) was performed by MR-guided wire localization (blue arrow). **(D)** The dynamic contrast-enhanced time–signal intensity curve (TIC) appeared as “wash-out type”. **(E)** The lesion was confirmed as low-grade intraductal carcinoma.

**Figure 2:**
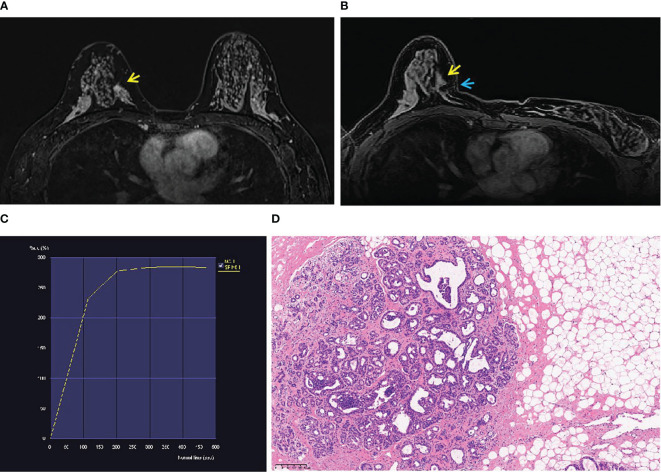
A 46-year-old woman’s physical examination. **(A)** A non-mass enhancement (yellow arrow) located in the inner quadrant of the right breast (BI-RADS 4A) was shown in T1-weighted fat-suppressed contrast-enhanced MRI. It was not detected by ultrasound or mammography. **(B)** The lesion (yellow arrow) was performed by MR-guided wire localization (blue arrow). **(C)** The dynamic contrast-enhanced time–signal intensity curve (TIC) appeared as “fast-plateau type”. **(D)** Postoperative pathological diagnosis was atypical hyperplasia.

Among the 131 enhanced breast lesions suspected in 126 women, 39 (29.77%) were malignant and 92 (70.23%) were benign. Among malignant lesions, intraductal carcinoma (**Figure 1**) is the most common (23/131, 17.56%). Among all the benign lesions, high-risk lesions (HRLs) are the major component that cannot be ignored, including atypical ductal hyperplasia (ADH) (39/131, 29.77%) (**Figure 2**), papilloma (12/131, 9.16%), sclerosing adenosis (2/131, 1.53%), and complex sclerosing adenopathy (9/131, 6.87%). The total proportion of HRLs is 47.32% (62/131). The details of pathological diagnosis classification are shown in **Table 2**.

**Table 2:** Pathological classification of 131 lesions.

Pathology	Number (%)
Malignancy	39 (29.77%)
Non-specific invasive carcinoma, grade I	2 (1.53%)
Non-specific invasive carcinoma, grade II	4 (3.05%)
Invasive lobular carcinoma	2 (1.53%)
Intraductal carcinoma, low grade	11(8.40%)
Intraductal carcinoma, intermediate grade	7(5.34%)
Intraductal carcinoma, high grade	5(3.82%)
Intraductal carcinoma with microinvasion	8 (6.11%)
Benignancy	92 (70.23%)
Atypical ductal hyperplasia	39 (29.77%)
Papilloma	12 (9.16%)
Complex sclerosing adenopathy	9 (6.87%)
Sclerosing adenosis	2 (1.53%)
Adenopathy	19 (14.50%)
Ductal epithelial hyperplasia	3 (2.29%)
Fibroadenoma	6 (4.58%)
Others	2 (1.53%)
Total	131

**Figure 3:**
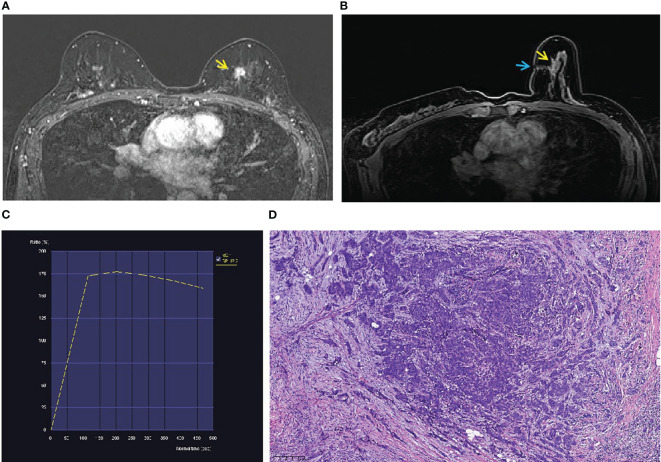
A 52-year-old woman with a family history of breast cancer. **(A)** An irregular mass (yellow arrow) located in the inner quadrant of the left breast (BI-RADS 4B) was shown in T1-weighted fat-suppressed contrast-enhanced MRI. It was not palpated and detected by ultrasound or mammography. **(B)** The mass (yellow arrow) was performed by MR-guided wire localization (blue arrow). **(C)** The dynamic contrast-enhanced time–signal intensity curve (TIC) appeared as “wash-out type”. **(D)** The lesion was confirmed as invasive carcinoma (grade II).

**Table 3** shows the MRI characteristics of all cases that were ultimately proven to be malignant and benign. Age, location, and the morphological characteristics on DCE-MRI are not statistically correlated with benign or malignant lesions (*p* > 0.05). The 131 lesions are composed of 80 non-mass enhancement (NME) lesions, 30 enhancement masses (EM), and 21 enhancement focuses (EF). However, malignancy rates were statistically correlated with background parenchymal enhancement (BPE) *χ* ^2^ = 24.979, *p* = 0.000).The minimal BPE group has the highest malignancy rate (17/39, 43.59%), while the ratio is the lowest in marked BPE (1/39, 2.56%). There was a statistical correlation between the TIC of the lesion and the malignant rate (*χ* ^2^ = 15.081, *p* = 0.001). A total of 38 lesions showed inflow curves (Type I), namely, 34 cases of benign lesion and only 4 cases of low-grade intraductal carcinoma. A total of 79 platform curves (Type II) were seen in benign (*n* = 53) and malignant lesions (*n* = 26). Nine of 14 washout curves (Type III) were malignant lesions (**Figure 3**), with the highest malignant rate (64.28%). All lesions were evaluated as BI-RADS Class 4 or above, including 77 BI-RADS 4A (benign, *n* = 64, 83.12%; malignancy, *n* = 13, 16.88%), 40 BI-RADS 4B (benign, *n* = 25, 62.50%; malignancy, *n* = 15, 37.50%), 12 BI-RADS 4C (benign, *n* = 3, 25.00%, malignancy, *n* = 9, 75.00%), and 2 BI-RADS 5 (malignancy, *n* = 2, 100%). There was statistical correlation between the BI-RADS category and the malignant rate (*χ* ^2^ = 23.719, P<0.001). The probability of malignancy increased with the BI-RADS category of the suspicious lesion.

**Table 3:** Clinical and MRI characteristics of 131 lesions confirmed as malignant and benign lesions.

	Malignancy	Benignancy	Total	*χ* ^2^	*p*
Age				4.779	0.092
≤40	7	34	41		
40–60	30	53	83		
≥60	2	5	7		
Location				0.51	0.475
Left breast	16	44	60		
Right breast	23	48	71		
BPE				24.979	0
Minimal	17	11	28		
Mild	14	26	40		
Moderate	7	24	31		
Marked	1	31	32		
Morphology				0.541	0.763
Focus	5	16	21		
Non-mass	24	56	80		
Mass	10	20	30		
TIC				15.081	0.001
Type I	4	34	38		
Type II	26	53	79		
Type III	9	5	14		
BI-RADS				23.719	< 0.001
4A	13	64	77		
4B	15	25	40		
4C	9	3	12		
5	2	0	2		

## Discussion

MRI offers the highest sensitivity of 94%–100% for detecting breast cancer, superior to mammography and US examination. However, the specificity of MRI is limited to 37%–72% (13, 16) due to the overlapping features of morphological image and hemodynamic features of DCE-MRI.

Because of the limited specificity of MR, if lesions are identified on MRI that are suspicious for malignancy, then a targeted US examination is advised to confirm that the lesion is indeed malignant. If there are suspicious malignant indications on US, the US-guided wire localization is the best option in clinical practice. Otherwise, MRI-guided biopsy must be performed. The efficacy of the second-look US is reported between 23% and 71%, with a malignancy detection rate of 15%–56% (17). A review reported that larger enhancing masses (MR BI-RADS 5 lesions) have a detection rate estimated between 25% and 62% on the second-look US; however, those non-mass enhancement or small foci (<10 mm) have a detection rate between 11% and 42% on the second-look US (18). Except for refusers, there were no abnormal findings in the second-look US of the remaining patients in our group.

MRI-guided breast biopsy is considered a necessary procedure in Western countries. However, there are some limitations in Asians due to its high cost and lack of professional procedure and expertise (19, 20). MRI-guided wire localization is an important intervention method, which is based on careful radiological analysis and accurate evaluation of BI-RADS Class 4 and above lesions. The overall malignant rate for MRI-guided wire localization ranges from 20% to 35% in European and American countries (21–24). The malignant rate of MRI-guided biopsy was 43% in 185 women with high risk of breast cancer according to Miah et al. in 2023 (25). Unfortunately, there are not many reports on this technology in Asian countries. Kuhl et al. (26) reported a malignancy rate of 55% with 95 lesions of 66 patients after MRI-guided wire localization resections. Cha et al. (27) reported a malignancy rate of 29.23% according to their analysis of 65 cases of wire localization. MRI-guided wire localization has gradually been applied in China in the past 10 years. In 2015, Wang et al. (13) performed MRI-guided wire localization on 44 Chinese female patients, and the confirmed malignant rate was 30.4%. The malignancy rate was 44% (in 75 lesions of 74 patients with MRI-guided wire localization) according to Wang et al. in 2020 (14). This study enrolled a total 131 MRI BI-RADS 4A and above lesions in 126 patients who had undergone wire localization, and the malignancy rate was 29.77% (39/131), which was within the malignancy risk range of previous reports and the American College of Radiology (ACR) BI-RADS^®^ Atlas 2013 benchmark for biopsy (15).

Most MRI-guided wire localization were benign (92/131, 70.23%) in our center, but the majority of them are HRLs (28–30). HRLs in the breast diagnosed by MRI-guided biopsy are a heterogeneous group of benign lesions, which may be confirmed to malignancy at subsequent surgical excision, and have some increased risks for the development of malignancy in the future. It is usually recommended that HRLs should be removed through surgical excision. Therefore, MRI-guided wire localization is an indispensable procedure before surgery.

DCE-MRI detects and evaluates breast lesions based on the morphological and hemodynamic changes. Lilly et al. (31) reported that there was no significant difference in the malignancy rate for NME lesions compared to mass lesions (*p* = 0.4). There was a statistical correlation between the enhancement pattern of lesions and the malignancy rate in the reports of Macura et al. (32), Dratwa et al. (33), and Moreno et al. (34) involving 541 MRI biopsy cases; they found that it was difficult to assess whether the lesion was benign or malignant based on simple enhancing morphology (NME, EM, or EF); however, the early rapid inflow (*p* = 0.038) and delayed outflow hemodynamics (*p* = 0.032) of DCE-MRI were significantly correlated with malignancy. Myers et al.’s (21) research findings suggested that the malignant rate was related to the “outflow” of TIC. In this study, the statistical correlation of benign or malignant lesions involved hemodynamics (TIC curve), rather than the morphological enhancement (EF, EM, and NME). The outflow of the TIC curve is more likely to be malignant lesion. Therefore, even if enhanced morphology cannot determine the pathological nature, we can combine with changes in hemodynamic “inflow”, “platform”, and “outflow” to improve the diagnostic efficiency of MRI.

The MRI BI-RADS data system defines a wide range of malignancy risk rates from 2% to 95% for BI-RADS 4 lesions (15). Biopsy is recommended for all of BI-RADS 4 lesions. However, both surgeons and patients hope to have a more accurate MRI assessment of lesions like US and x-ray in clinical practice. Maltez de Almeida et al. (35) found that BI-RADS 4C lesions have the highest likelihood of malignancy (103 MRI BI-RADS 4C lesions in 83 patients). Dratwa et al. (33) analyzed 208 lesions of BI-RADS 4B and above, and found that the probability of malignancy increased with the BI-RADS category of the lesion. Chevrie et al. (17) also reported that the PPV of breast lesions in MR-guided biopsy increased with the BI-RADS category of the lesions. Thus, we classified the lesions into BI-RADS 4A, 4B, or 4C category based on DCE-MRI enhancement morphology and the TIC curve. In our study, there were 77 BI-RADS 4A lesions (58.77%, 77/131), with a malignant rate of 16.88% (13/77); 40 lesions of 4B (30.53%,40/131), with a malignant rate of 37.50% (15/40); 12 lesions of 4C (9.16%, 12/131), with a malignant rate of 75.00% (9/12); and 2 lesions of 5 category, with a malignancy rate of 100%. There was a statistical correlation between the MRI BI-RADS category and pathological malignancy (*χ* ^2 ^= 23.719, P<0.001). The malignant rate in MRI-guided wire localization increases with the increase of BI-RADS category. Similar results were also reported by Wang et al. in China (14).

BPE may bring certain challenges for breast DCE-MRI. Owing to the non-visualization of the target lesion or changed MRI findings, which may indicate BPE, MRI-guided breast biopsy may be canceled. The rate of MRI-guided biopsy cancellation was nearly 8%–13% according to previous researches (27, 36). Brennan et al. (37) found that severe and moderate BPE may lead to a higher cancellation rate of MRI biopsy. The reasons for biopsy cancellations include a hormonal false effect, which can affect vascularity and BPE, as well as focal inflammatory or fibrocystic changes that had dissipated at the time of the scheduled biopsy. To avoid the effect of hormone fluctuations on breast BPE, we performed MRI-guided wire localization and the previous MRI within the same menstrual period.

BPE has been associated with risk factors for breast cancer (38, 39). The moderate/marked compared to minimal/mild BPE was significantly associated with breast cancer among premenopausal women; however, among postmenopausal women, the data did not reach statistical significance to draw any firm conclusions (38). The results of Cha et al. (27) showed that BPE did not differ between the malignant and benign lesions. Myers et al. (21) reported that BPE was more significant in the malignant lesion confirmed by MRI biopsy. There was a statistical difference between the breast BPE of benign and malignant lesions, according to Moreno et al. (34). Lilly et al. (31) reported that the highest malignancy rate (37%) was in the minimal BPE group, while no malignancies were identified with moderate to marked BPE in women. Similarly, the mild BPE group had the highest malignancy rate (17/39, 43.59%), and the proportion of significant BPE was the lowest (1/39, 2.56%) in our study. Analyzing the reasons for this result, we consider that it cannot completely remove the diagnostic interference caused by moderate or even marked BPE, even though subtraction technology has been applied in diagnostic MRI scans. It is undeniable that the increased BPE may increase the false-positive rate of MRI and MRI-guided intervention. The more significant the BPE, the more suspicious lesions may enter the biopsy, while the actual malignancy rate is actually lower than that of the mild BPE group. Therefore, we believe that any suspicious findings, with an assessment of BI-RADS 4A or above detected upon MRI only, should be located and biopsied regardless of BPE.

For suspicious BI-RADS 4A and above lesions displayed only on MRI, vacuum-assisted biopsy (VAB) and core needle biopsy (CNB) are both commonly used methods. Adequate and accurate sampling is key to obtaining reliable pathological results from MRI-guided biopsy. Small lesions are a major difficulty in biopsy. If the volume of the biopsy sample is deficient or the sampling location is not accurate, it may lead to an untruthful pathological diagnosis result. Some HRLs that have been diagnosed through MRI-guided biopsy may be upgraded to malignancy at surgical excision (28, 29, 40). Compared to wire-guided localization (WGL), MRI-guided biopsy cannot avoid the above-mentioned deficiency and obstacles. MRI-guided breast biopsy has become an essential clinical skill for doctors in Western European countries (5, 6). For institutions that are unable to perform (or have experienced difficulty in performing) breast MRI-guided biopsy, MRI-guided wire localization is a good alternative method, which is very important to ensure complete removal of suspected malignant lesions with negative margins (41). It is undeniable that there is a lack of enough clinical experience in conducting MRI-guided breast biopsy in China. When the presence of suspicious lesions was suggested in breast MRI, most female Chinese patients were always very anxious and required to undergo surgical resection followed by MRI-guided wire localization. In addition to obtaining histological diagnosis, MRI-guided wire localization is also useful for complete resection of suspected malignant lesions. It can effectively avoid the dilemma of insufficient puncture tissue, or significant differences between puncture and postoperative pathology.

In addition, radio-guided surgery is an approach that assists the surgical excision of non-palpable breast lesions by using a gamma probe to detect a preinserted marker (42). It is implemented in two forms as follows: radioactive seed localization (RSL) is based on the detection of a small 125-iodine seed, while radioactive occult lesion localization (ROLL) relies on the identification of a preinjected radiocolloid (99m Technetium) (43). RSL has a wider application in x-ray than MR, which is considered a standard approach in the United States, Canada, and the Netherlands (44). RSL was as equally reliable as WGL (45, 46). However, the seeds of RSL are not approved for use in other countries including China. ROLL has a wide range of applications, including x-ray, US, and MR, which is a popular technique in some countries (Turkey, Australia, and Latin America) but remains unknown in others. A review showed comparable rates of successful excision of the target lesion between the technique and WGL (43). Some reported slightly higher costs of RSL for each patient than WGL (47), while others showed a lower average cost per patient for RSL (48). The mean cost of ROLL was found to be slightly more expensive than WGL in two RCTs comparing costs (49).

This study enrolled a total of 131 lesions by MRI-guided wire localization and resection in 126 patients. Although the sample quantity is larger than previous reports in China, we cannot deny that it still has certain limitations. Our study is limited by a retrospective study design and a single institution. It is undeniable that selection bias and unique practice influence in cancer hospital. However, the success rate of MRI-guided wire localization in this group was 100%, and all lesions were confirmed by pathological diagnosis, which was helpful for further follow-up technology promotion and management in multiple centers. Therefore, MRI-guided wire localization is an effective and safe method, which is of great significance for improving the early diagnosis and treatment of Chinese women with breast cancer.

## Data availability statement

The raw data supporting the conclusions of this article will be made available by the authors, without undue reservation.

## Ethics statement

The studies involving humans were approved by Medical Ethics Committee of the Shaanxi Provincial Cancer Hospital Affiliated to Medical School Xi’an Jiao tong University. The studies were conducted in accordance with the local legislation and institutional requirements. According to the approval of the Ethics Committee of Shaanxi Cancer Hospital, informed consent of patients has been exempted in this study.

## Author contributions

JM: Writing – review & editing, Writing – original draft, Validation, Methodology, Investigation, Formal analysis, Data curation. LH: Investigation, Methodology, Formal analysis, Data curation, Writing – original draft. XL: Supervision, Project administration, Writing – review & editing. BY: Resources, Methodology, Writing – review & editing, Formal analysis, Data curation. QD: Resources, Methodology, Writing – review & editing, Data curation. HG: Resources, Methodology, Data curation, Writing – review & editing. JZ: Software, Writing – review & editing, Validation. CS: Software, Supervision, Methodology, Writing – review & editing. QY: Software, Writing – review & editing, Supervision, Methodology. YW: Conceptualization, Project administration, Investigation, Funding acquisition, Writing – review & editing, Supervision.
